# Interaction between NO and COX pathways modulating hepatic endothelial cells from control and cirrhotic rats

**DOI:** 10.1111/j.1582-4934.2012.01563.x

**Published:** 2012-09-26

**Authors:** Eugenio Rosado, Aina Rodríguez-Vilarrupla, Jorge Gracia-Sancho, Montserrat Monclús, Jaume Bosch, Joan-Carles García-Pagán

**Affiliations:** Hepatic Hemodynamic Laboratory Liver Unit IMDIM Hospital Clínic, Institut d'Investigacions Biomèdiques August Pi i Sunyer (IDIBAPS) and Ciberehd University of BarcelonaBarcelona, Spain

**Keywords:** cyclooxygenase, nitric oxide, superoxide, portal hypertension, endothelium

## Abstract

Reduced intrahepatic nitric oxide (NO) bioavailability and increased cyclooxygenase-1 (COX-1)-derived vasoconstrictor prostanoids modulate the hepatic vascular tone in cirrhosis. We aimed at investigating the reciprocal interactions between NO and COX in the hepatic endothelium of control and cirrhotic rats. NO bioavailability (DAF-FM-DA staining), superoxide (O_2_^−^) content (DHE staining), prostanoid production (PGI_2_ and TXA_2_ by enzyme immunoassays) as well as COX expression (Western Blot), were determined in hepatic endothelial cells (HEC) from control and cirrhotic rats submitted to different experimental conditions: COX activation, COX inhibition, NO activation and NO inhibition. In control and cirrhotic HEC, COX activation with arachidonic acid reduced NO bioavailability and increased O_2_^−^ levels. These effects were abolished by pre-treating HEC with the COX inhibitor indomethacin. In control, but not in cirrhotic HEC, scavenging of O_2_^−^ by superoxide dismutase (SOD) incubation partially restored the decrease in NO bioavailability promoted by COX activation. NO supplementation produced a significant and parallel reduction in PGI_2_ and TXA_2_ production in control HEC, whereas it only reduced TXA_2_ production in cirrhotic HEC. By contrast, in control and cirrhotic HEC, NO inhibition did not modify COX expression or activity. Our results demonstrate that NO and COX systems are closely interrelated in HEC. This is especially relevant in cirrhotic HEC where COX inhibition increases NO bioavailability and NO supplementation induces a reduction in TXA_2_. These strategies may have beneficial effects ameliorating the vasoconstrictor/vasodilator imbalance of the intrahepatic circulation of cirrhotic livers.

## Introduction

In cirrhotic livers, increased resistance to portal blood flow is the primary factor in the pathophysiology of portal hypertension [1, 2]. This increase in intrahepatic resistance is determined by architectural alterations of the liver as well as by a dynamic component, which is due to an increased production of COX-1-derived vasoconstrictors, such as thromboxane (TXA_2_) [3, 4], in the setting of an insufficient availability of the vasodilator NO [5, 6].

Previous studies from our group have demonstrated that HEC, which overexpress COX-1 [7], are an important source of vasoconstrictor prostanoids, mainly TXA_2_ [8]. In addition, COX contributes to the increased oxidative stress found in cirrhotic rat livers [9]. On the other hand, reduced NO bioavailability in cirrhotic rat livers is not only attributable to a decreased nitric oxide synthase activity but also to an increased scavenging by superoxide anions (O_2_^−^) [10]. Particularly, NO availability is modulated by O_2_^−^ in HEC [9]. As expected, the beneficial effects of COX inhibitors improving endothelial dysfunction in cirrhotic rat livers was attenuated when NO synthesis was previously inhibited [3, 11].

In endothelial cells from different vascular beds, there are evidence supporting direct and indirect interactions between NO and COX systems [12–14] as NO could positively or negatively modulate COX activity and modify eicosanoid production [15–18]. Conversely, COX-derived prostanoids, such as TXA_2_, are known to modulate NO-synthase activity, downregulating its phosphorylation and decreasing its activity [19]. Such interactions may also be present in the cirrhotic liver; specifically we hypothesize that NO and COX systems are interrelated in HEC. The current study was aimed at investigating possible reciprocal modulation between NO and COX pathways in the hepatic endothelium of control and cirrhotic rats.

## Materials and methods

### Induction of cirrhosis by carbon tetrachloride

Male Wistar rats weighing 50–75 g underwent inhalation exposure to Carbon Tetrachloride (CCl_4_). Phenobarbital (0.3 g/l) was added to the drinking water as previously described [3]. A high yield of micronodular cirrhosis was obtained after approximately 12–15 weeks of CCl_4_ inhalation. When the cirrhotic rats developed ascites, administration of phenobarbital was stopped, and the subsequent experiments were performed 1 week later. Control animals received only phenobarbital. The animals were kept in environmentally controlled animal facilities at the Institut d'Investigacions Biomèdiques August Pi i Sunyer. All experiments were approved by the Laboratory Animal Care and Use Committee of the University of Barcelona and were conducted in accordance with *Guide for the Care and Use of Laboratory Animals* (National Institutes of Health, NIH Publication 86-23, revised 1996).

### Isolation and culture of hepatic endothelial cells

Hepatic endothelial cells were isolated from control and cirrhotic rats as previously described [9]. Briefly, after collagenase perfusion of the livers and isopycnic sedimentation of the resulting dispersed cells through a two-step density gradient of Percoll, pure monolayer cultures of HEC were established by selective attachment on a substrate of rat tail collagen type I. Afterwards, cells were cultured in Roswell Park Memorial Institute (RPMI)-1640 and studies were performed on cells from the first passage, 12 hrs after their isolation, to preserve their typical phenotype [20].

### Experimental protocols

#### Effects of COX activity on NO bioavailability and O_2_^−^ production in control and cirrhotic HEC

Hepatic endothelial cells isolated from control and cirrhotic rats were pre-incubated for 15 min. with vehicle or with the non-selective COX inhibitor, indomethacin (10 μM). Then, arachidonic acid (AA, 40 μM) or its vehicle (ethanol 0.1%) was added. After 20 min., *in situ* NO bioavailability and O_2_^−^ levels were assessed with 4-amino-5-methylamino-2′,7′-difluorofluorescein diacetate (DAF-FM-DA; Molecular Probes Inc., Eugene, OR, USA) and dihydroethidium (DHE; Molecular Probes Inc.), respectively, as described below.

To characterize whether O_2_^−^ derived from COX modulates NO bioavailability, a different group of HEC from control or cirrhotic rats were treated with vehicle (phosphate-buffered saline; PBS), with AA alone or with AA plus the superoxide scavenger, superoxidedismutase (SOD: 300U). This dose of SOD has been shown to markedly attenuate the marked increase in O_2_^−^ produced by the SOD inhibitor, diethyldithiocarbamate [9]. In parallel, control HEC were treated with the NO synthase (NOS) inhibitor *N*_ω_-Nitro-l-arginine methyl ester hydrochloride, L-NAME (1.5 mM) as a negative control. Vascular endothelial growth factor (VEGF, 40 ng/ml) was added to cirrhotic HEC in order to prime NO production. Fluorescence images were obtained every 2 min. for 40 min. with a laser scanning confocal microscope.

#### Effects of NO on COX activity in control and cirrhotic HEC

*Effects of NO supplementation on COX activity in control and cirrhotic HEC*: HEC isolated from control and cirrhotic rats were treated with the exogenous NO donor, sodium nitroprusside (SNP, 25 μM) or its vehicle (PBS) for 1 hr, and then incubated with AA for 20 min. Culture media samples were collected, stored at −80°C and assayed for prostanoid levels. Cells were lysed and processed for Western blot analysis as previously described [21].

*Effects of NO inhibition on COX activity in control and cirrhotic HEC*: HEC isolated from control and cirrhotic rats were pre-incubated with the NOS inhibitor L-NAME or its vehicle (PBS) for 1 hr. Then, the calcium ionophore A23187 (2 μM), which by increasing calcium levels activates both COX and NOS, or its vehicle (Dimethyl sulfoxide 0.01%) were added. After 1 hr of treatment, culture media samples were collected and stored at −80°C until assayed for prostanoid levels. Cells were lysed and processed for Western blot analysis.

### Measurement of NO levels and O_2_^−^ content in HEC

*In situ* NO levels or O_2_^−^ levels in HEC were assessed with 4-amino-5-methylamino-2′,7′-difluorofluorescein diacetate (DAF-FM-DA; Molecular Probes Inc.) or with the oxidative fluorescent dye dihydroethidium (DHE; Molecular Probes Inc.) as described [9, 22].

Briefly, isolated HEC were washed in RPMI-1640 without phenol red and loaded with DAF-FM-DA (10 μM for 20 min. at 37°C) or DHE (10 μM for 20 min. at 37°C). Then, HEC were rinsed three times with PBS, kept in the dark, and maintained at 37°C with a warm stage on a laser scanning confocal microscope (model TCS-SL DMIRE2; Leica, Wetzlar, Germany). Fluorescence images were obtained with a 488-nm (excitation) and 505- to 530-nm (emission) filter set for DAF-FM-DA, and 610-nm (emission filter) set for DHE with a 40 × 1.3 oil objective. Quantitative analysis was obtained by averaging of the peak relative fluorescent intensity (optical density arbitrary units) of each confocal microscope image (Image J 1.43m software, National Institutes of Health) and normalization of the fluorescent result by the total number of cultured cells counted from each corresponding digitalized phase contrast microscope image.

### Analysis of prostanoids

Prostacyclin (PGI_2_) and TXA_2_ were quantified in duplicate as their stable metabolites, 6-keto PGF_1α_ and TXB_2_, respectively, as previously described [8] by using enzyme immunoassay kits. All assays contained media controls to exclude any effect of the reagents on the immunoassays.

### Western blot analysis of COX-1, prostacyclin synthase and thromboxane synthase protein expression in HEC

Aliquots from each sample containing equal amounts of protein (10 μg) were run on a sodium dodecyl sulfate-polyacrylamide gel and transferred to a nitrocellulose membrane. After the transfer, the blots were blocked for 1 hr and were probed with a mouse anti-COX-1 antibody (5 μg/ml), rabbit anti-prostacyclin synthase (PGIS) (2 μg/ml) or rabbit anti-thromboxane synthase (TXAS) (1 μg/ml) overnight at 4°C followed by incubation with their associated horseradish peroxidase–conjugated secondary antibody (1:10,000; Stressgen Victoria, British Columbia, Canada) for 1 hr at room temperature. Blots were revealed by chemiluminiscence. Protein expression was determined by densitometric analysis with the Science Laboratory Image Gauge (Fuji Photo Film GmbH, Düsseldorf, Germany). After stripping, blots were assayed for glyceraldehyde 3-phosphate dehydrogenase (GAPDH; Santa Cruz Biotechnology, Santa Cruz, CA, USA) expression as a standardization of the sample loading. Quantitative densitometric values of all proteins were normalized to GAPDH.

### Drugs and reagents

AA, enzyme immunoassays kits, COX-1, PGIS and TXAS antibodies were obtained from Cayman Chem Co (Tallin, Estonia). Collagen type I was from Invitrogen (El Prat de Llobregat, Barcelona, Spain). Collagenase was from Roche Diagnostics (Mannheim, Germany). Percoll was from Amersham Biosciences (Uppsala, Sweden). Reagents for cell culture were provided by Biological Industries Ltd (Kibbutz Beit Haemek, Israel). L-NAME, A23187, SNP, indomethacin and other chemical compounds were purchased from Sigma-Aldrich (Tres Cantos, Madrid, Spain).

### Statistical analysis

Statistical analysis was performed using the SPSS 16.0 for Windows statistical package (SPSS Inc., Chicago, IL, USA). All results are expressed as mean ± S.E.M. Comparisons between groups were performed with the Student's *t*-test or Mann–Whitney *t*-test for unpaired data or anova for repeated measures when adequate. Differences were considered significant at a *P*-value <0.05.

## Results

### Effects of COX activity on NO bioavailability and O_2_^−^ production in control and cirrhotic HEC

In the first set of experiments, we investigated whether activation or inhibition of COX pathway modulates NO bioavailability.

#### COX activation reduces NO bioavailability and increases oxidative stress in control and cirrhotic HEC

**Fig 1:**
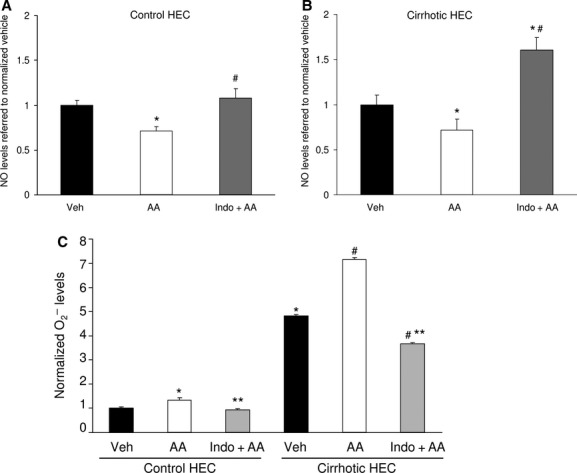
Effects of COX activity on nitric oxide (NO) bioavailability and superoxide (O_2_^−^) levels in hepatic endothelial cells (HEC) from control and cirrhotic rats. Fluorescent detection of intracellular NO bioavailability by 4-amino-5-methylamino-2′,7′difluorofluorescein diacetate (DAF-FM-DA) staining and O_2_^−^ by dihydroethidium (DHE) staining in HEC. Arachidonic acid (AA) administration promoted a significant decrease in NO bioavailability in control (A) and cirrhotic (B) HEC which was prevented by coincubation with indomethacin. Besides, in cirrhotic HEC, indomethacin treatment increased NO bioavailability above baseline values. The fluorescence intensity of DAF-FM-DA in arbitrary units was normalized by the total number of cells. The data shown are from 5296 individual vehicle (Veh) control HEC, 5322 AA control HEC, 3124 indomethacin+AA (Indo+AA) control HEC, 1082 individual Veh cirrhotic HEC, 1280 AA cirrhotic HEC, and 1228 Indo+AA cirrhotic HEC obtained from three independent experiments. The mean NO levels of HEC treated with vehicle was considered one, either for control or cirrhotic rats (*P < 0.05 *versus* Veh, ^#^*P* < 0.05 *versus* AA). (C) O_2_^−^ was significantly higher in HEC from cirrhotic than in control rats. Arachidonic acid (AA) promoted a significant increase in O_2_^−^ in control and cirrhotic HEC which was prevented by indomethacin. In cirrhotic HEC, indomethacin treatment decreased O_2_^−^ below baseline values. The fluorescence intensity of DHE in arbitrary units was normalized by the total number of cells. The data shown are from 5642 individual Veh control HEC, 4713 AA control HEC, 4682 Indo+AA control HEC, 2616 individual Veh cirrhotic HEC, 1399 AA cirrhotic HEC, and 1101 Indo+AA cirrhotic HEC obtained from four independent experiments (*P < 0.05 *versus* control Veh, ^#^*P* < 0.05 *versus* cirrhotic Veh, **P < 0.05 *versus* its own AA-condition).

To investigate the effects of COX activation on NO bioavailability, HEC from control and cirrhotic rats were treated with AA. AA administration promoted a significant and marked decrease in NO bioavailability both in HEC from control (Fig. 1A) and from cirrhotic rats (Fig. 1B).

COX activation also promoted a significant increase in O_2_^−^ content in HEC from control or cirrhotic rats, being this increase much higher in the later (Fig. 1C).

#### COX inhibition increases NO bioavailability and reduces oxidative stress in control and cirrhotic HEC

To further explore the role of COX modulating the NO–O_2_^−^ relationship, COX was inhibited before AA administration. Indomethacin prevented the decrease in NO bioavailability produced by AA in HEC from both control (Fig. 1A) and cirrhotic rats (Fig. 1B). Remarkably, in cirrhotic HEC, indomethacin treatment increased NO bioavailability above baseline values, supporting the role of COX activation reducing NO bioavailability.

Furthermore, COX inhibition prevented the increase in O_2_^−^ content caused by AA in HEC from control and cirrhotic rats (Fig. 1C). In cirrhotic HEC, which displayed significantly higher levels of O_2_^−^ in comparison with control HEC (Fig. 1C), indomethacin decreased O_2_^−^ levels below baseline values, suggesting that COX is a source of O_2_^−^ in cirrhotic HEC.

#### Effects of COX-derived O_2_^−^ modulating NO bioavailability in HEC

To assess whether O_2_^−^ is the main determinant of the reduction of NO bioavailability caused by COX activation, we assessed NO bioavailability in control and cirrhotic HEC incubated with AA or with AA plus the superoxide scavenger, SOD.

**Fig 2:**
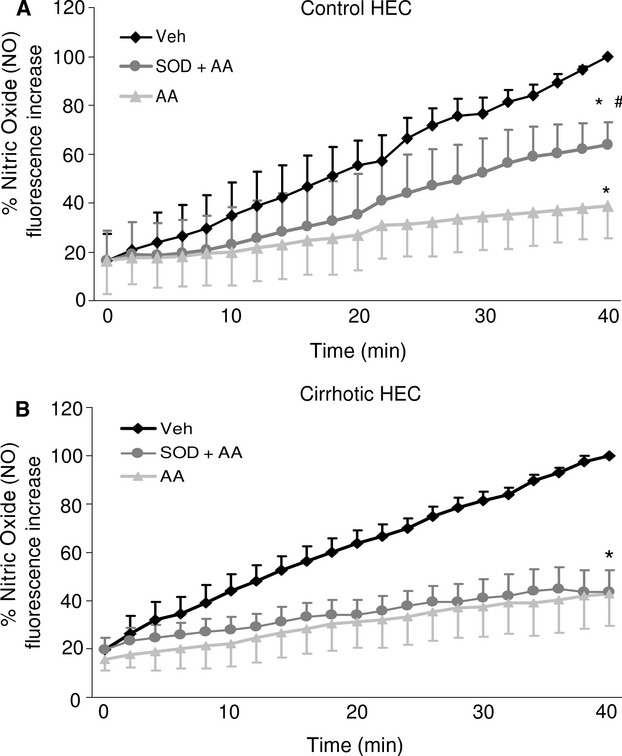
Effects of COX activation on NO production in control and cirrhotic hepatic endothelial cells (HEC). Time dependent increase in DAF-FM-DA fluorescence as a marker of intracellular NO bioavailability. (A) In control HEC, AA incubation induced a significant decrease in NO bioavailability which was partially restored by coincubation with SOD (*P < 0.05 *versus* vehicle, ^#^*P* < 0.05 *versus* AA). (B) In cirrhotic HEC, AA incubation induced a significant decrease in NO bioavailability which was not restored by coincubation with SOD (*P < 0.05 *versus* vehicle). The fluorescence intensity of DAF-FM-DA was normalized by the total number of cells of each experimental condition. The data shown are from 409 individual vehicle control HEC, 317 AA control HEC and 256 SOD+AA control HEC, obtained from three independent experiments and 701 vehicle cirrhotic HEC, 767 AA cirrhotic HEC and 750 SOD+AA cirrhotic HEC obtained from five independent experiments.

In control HEC, AA administration promoted a significant decrease in NO bioavailability. This effect was attenuated when O_2_^−^ was scavenged by SOD coincubation, indicating that O_2_^−^ was reducing NO bioavailability (Fig. 2A).

In HEC from cirrhotic rats, AA administration promoted a marked decrease in NO production. However, contrary to control HEC, SOD coincubation did not attenuate the decrease in NO bioavailability suggesting that, in cirrhotic HEC, O_2_^−^ was not a major determinant of decreased NO bioavailability after COX activation (Fig. 2B).

### Effects of NO on COX pathway in control and cirrhotic HEC

In a second series of experiments, HEC from control and cirrhotic rats were incubated with a NO synthase inhibitor or with a NO donor to elucidate whether variations in NO levels modulate COX pathway.

#### NO supplementation induces a marked reduction of COX activity in control and cirrhotic HEC

**Fig 3:**
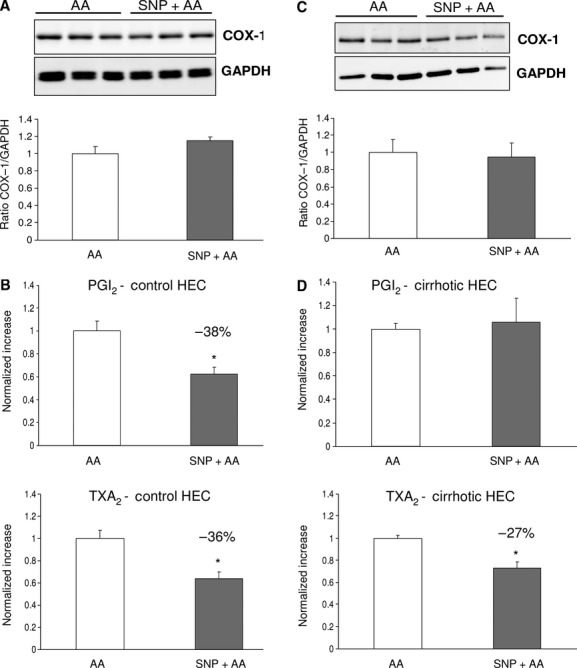
Effects of NO supplementation on COX pathway in control and cirrhotic hepatic endothelial cells (HEC). (A) Representative Western blot and analysis of COX-1 from control HEC treated with sodium nitroprusside (SNP) or its vehicle in the presence of AA. Densitometry quantification in arbitrary units, normalized to glyceraldehyde 3-phosphate dehydrogenase (GAPDH), showed no differences between both groups. (*n* = 5 per group). (B) Prostacylin (PGI_2_) and Thromboxane (TXA_2_) production by HEC from control rats stimulated with AA in the presence or absence of the exogenous NO donor SNP (*n* = 6 per group). In control HEC, NO supplementation produced a significant and similar decrease in PGI_2_ (−38%) and TXA_2_ (−36%). (*P < 0.05 *versus* AA). (C) Representative Western blot and analysis of COX-1 from cirrhotic HEC treated with sodium nitroprusside (SNP) or its vehicle in the presence of AA. Densitometry quantification in arbitrary units, normalized to glyceraldehyde 3-phosphate dehydrogenase (GAPDH), showed no differences between both groups. (*n* = 7 per group). (D) Prostacylin (PGI_2_) and Thromboxane (TXA_2_) production by HEC from cirrhotic rats stimulated with AA in the presence or absence of the exogenous NO donor SNP (*n* = 6 per group). In cirrhotic HEC, NO supplementation resulted in a significant reduction in TXA_2_ production (−27%) without significant changes in PGI_2_ (*P < 0.05 *versus* AA).

In control HEC, NO supplementation did not modify COX-1 protein expression (Fig. 3A) or the basal level of PGI_2_ and TXA_2_ (data not shown). As expected, AA administration induced a significant increase in PGI_2_ and TXA_2_ [8] that was blunted when HEC were pre-incubated with the NO donor SNP (Fig. 3B).

In cirrhotic HEC, NO supplementation did not modify COX-1 protein expression (Fig. 3C). However, it significantly reduced TXA_2_ levels without significant changes in PGI_2_ production (Fig. 3D), suggesting that NO supplementation could partially restore the pathological disequilibrium in the PGI_2_/TXA_2_ ratio described in cirrhotic HEC.

#### NO inhibition does not modify COX pathway in control and cirrhotic HEC

**Fig 4:**
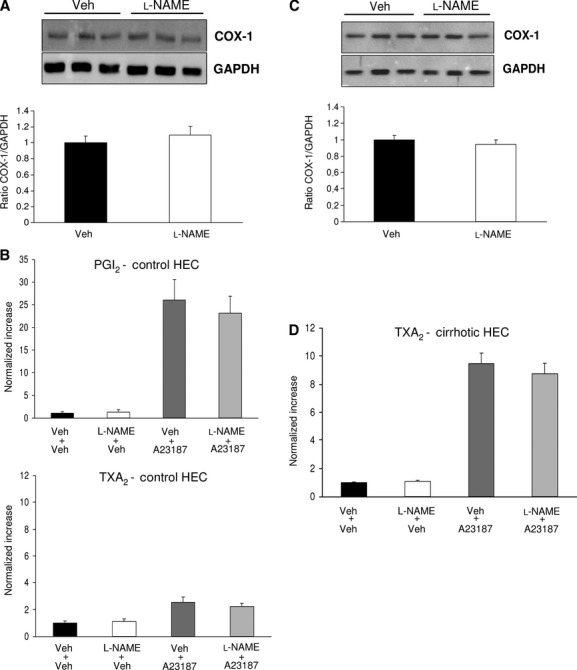
Effects of NO inhibition on COX pathway in control and cirrhotic hepatic endothelial cells (HEC). (A) Representative Western blot and analysis of COX-1 from control HEC treated with vehicle (Veh) or with L-NAME. Densitometry quantification in arbitrary units, normalized to glyceraldehyde 3-phosphate dehydrogenase (GAPDH), showed no differences between both groups (*n* = 7 per group). (B) Prostacyclin (PGI_2_) and Thromboxane (TXA_2_) production in control HEC treated with Veh or L-NAME in the presence of the calcium ionophore A23187 or its Veh (*n* = 6 per group). The mean prostanoid levels of HEC treated with Veh+Veh was considered one. NO inhibition did not modify either basal or A23187-induced prostanoid (PGI_2_ and TXA_2_) production. (C) Representative Western blot and analysis of COX-1 from cirrhotic HEC treated with vehicle (Veh) or with L-NAME. Densitometry quantification in arbitrary units, normalized to glyceraldehyde 3-phosphate dehydrogenase (GAPDH), showed no differences between both groups (*n* = 9 per group). (D) Thromboxane (TXA_2_) production by cirrhotic HEC treated with Veh or L-NAME in the presence of A23187 or its Veh (*n* = 9 per group). The mean prostanoid levels of HEC treated with Veh+Veh was considered one. NO inhibition did not modify either basal or A23187 induced TXA_2_ production.

As expected, HEC from control rats incubated with the nitric oxide synthase inhibitor L-NAME, exhibited a significant reduction in NO bioavailability (data not shown). However, L-NAME pre-incubation did not modify COX-1 expression (Fig. 4A) or activity, as evaluated by prostanoid production (Fig. 4B). In HEC from control rats, A23187 treatment, which simultaneously activate COX and NOS pathways, produced a marked release of PGI_2_ and TXA_2_ that was not modified by NO inhibition.

Similarly, in HEC isolated from cirrhotic rats, NO inhibition did not modify COX-1 protein expression (Fig. 4C) or basal TXA_2_ production, the main prostanoid produced by cirrhotic HEC. HEC from cirrhotic rats incubated with A23187 exhibited a significant increase in TXA_2_ which was significantly higher than that observed in control HEC and that was not modified by NO inhibition (Fig. 4D).

### PGIS and TXAS expression in control and cirrhotic HEC

**Fig 5:**
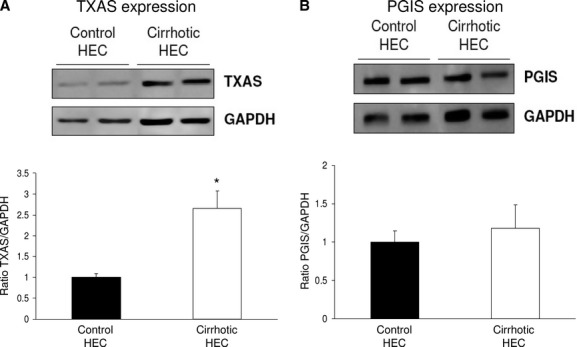
Prostacyclin and Thromboxane synthase protein expression in control and cirrhotic hepatic endothelial cells (HEC). (A) Representative Western blot and analysis of prostacyclin synthase (PGIS) from control and cirrhotic HEC. Densitometry quantification in arbitrary units, normalized to glyceraldehyde 3-phosphate dehydrogenase (GAPDH), showed no differences between both groups (*n* = 4 per group). (B) Representative Western blot and analysis of thromboxane synthase (TXAS) from control and cirrhotic HEC. Densitometry quantification in arbitrary units, normalized to glyceraldehyde 3-phosphate dehydrogenase (GAPDH), showed a significant increase in TXAS in cirrhotic HEC (*n* = 4 per group) (*P < 0.05 *versus* control HEC).

A third set of experiments were aimed at elucidating the main prostanoid synthase involved in the observed NO-COX interactions. For that purpose, PGIS and TXAS protein expression were determined in HEC from control and cirrhotic rats. Cirrhotic HEC exhibited a significantly higher TXAS expression compared with control HEC (Fig. 5A) whereas PGIS expression remained unchanged (Fig. 5B).

## Discussion

Hepatic endothelial cells play a major role regulating intrahepatic vascular tone by the release of vasoactive substances that diffuse to hepatic stellate cells and possibly to other contractile structures causing their relaxation or constriction [23]. Among these vasoactive mediators, we and others have demonstrated a major role for NO and COX-derived prostanoids. In normal livers, HEC have a vasodilator phenotype with predominant production of NO and PGI_2_ and reduced amounts of TXA_2_. By contrast, cirrhotic livers exhibit endothelial dysfunction characterized by a change to a vasoconstrictor phenotype. In that regard, HEC from cirrhotic rats have been shown to produce large amounts of the COX-derived vasoconstrictor prostanoid TXA_2_ [8], while NO is markedly reduced [9].

A reciprocal regulation between NO and COX-derived products, such as TXA_2_, has been demonstrated in endothelial cells from different vascular beds [12, 24]. However, the interactions between these molecular pathways in the hepatic endothelium have not been investigated.

The present study shows, for the first time in HEC, that both systems are reciprocally related. Indeed, COX products are able to modify NO bioavailability, whereas NO content modulates COX-derived prostanoid production.

Our study clearly demonstrates that COX activation induces a reduction in intracellular NO bioavailability in both control and cirrhotic HEC. Remarkably, in cirrhotic HEC, COX activation further reduces the already low NO bioavailability. COX activation markedly increases the production of TXA_2_ and PGI_2_ but also leads to an increase in O_2_^−^. TXA_2_ has been shown to reduce NO synthase activity in endothelial cells through a receptor-mediated mechanism [19, 25]. In addition, we have recently demonstrated in HEC that O_2_^−^ is able to interact with NO reducing its bioavailability [9]. Thus, COX activation could modulate NO bioavailability through the production of prostanoids or O_2_^−^. Our results suggest that the COX-derived product responsible for this decrease in NO availability is different in control and cirrhotic HEC. In control HEC, abolition of O_2_^−^ content with SOD partially restored NO availability, indicating that COX-derived O_2_^−^ was modulating, at least in part, NO availability. By contrast, in cirrhotic HEC, SOD administration was not able to restore at all the reduction in NO caused by COX activation. It may be argued that the used dose of SOD may have not sufficiently scavenged the higher increase in O_2_^−^ observed in cirrhotic livers in comparison with controls. However, this is unlikely because the same dose of SOD has been previously shown to markedly attenuate the huge increase in O_2_^−^ promoted in an experimental model of induced oxidative stress caused by direct SOD inhibition [9]. Altogether this suggests that, in cirrhotic HEC, COX-derived O_2_^−^ is not the main determinant in the reduction of NO bioavailability. Although the exact mechanism remains conjectural, we propose that COX-derived TXA_2_, and not O_2_^−^, contributes to the reduced NO bioavailability found in cirrhotic HEC. This is supported by the fact that the production of TXA_2_, as well as TXAS expression, is markedly increased in HEC isolated from cirrhotic but not from control livers, where PGI_2_ production predominates [8].

**Fig 6:**
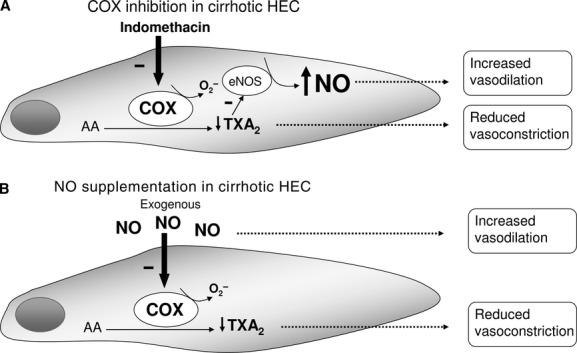
Suggested molecular mechanisms between NO and COX pathways in cirrhotic hepatic endothelial cells (HEC). (A) NO supplementation to cirrhotic HEC inhibits COX activity by reducing the vasoconstrictor prostanoid TXA_2_. This effect leads the cell to shift from a vasoconstrictor to a more vasodilatory phenotype suggesting that NO could promote hepatic vasodilation and reduce the exacerbated vasoconstriction in cirrhosis. (B) COX inhibition to cirrhotic HEC increases NO bioavailability. Therefore, COX inhibition would contribute to improve hepatic vascular tone, not only by reducing TXA_2_ production but also by increasing NO production.

The negative effect of COX activation on NO availability is further supported by our findings that COX inhibition restored NO bioavailability in control HEC, and that it even increased NO levels above baseline values in cirrhotic HEC. Therefore, our data strongly suggest that in cirrhotic HEC COX inhibition would contribute to improve hepatic vascular tone, not only by reducing TXA_2_ production but also by increasing NO production (Fig. 6A).

Another important aspect of our study was to assess whether NO availability could influence prostanoid production in control and cirrhotic HEC. Interestingly, our data demonstrate that NO supplementation is able to inhibit COX activity, as shown by the observed reduction in prostanoid production, without modifying COX expression. The effect of NO in COX activity was different in HEC from control or from cirrhotic rats. In control HEC, NO supplementation reduced the vasodilator PGI_2_ and the vasoconstrictor TXA_2_, thus maintaining the predominant vasodilator phenotype. By contrast, in cirrhotic HEC, NO supplementation significantly reduced TXA_2_ levels, the main prostanoid produced by these cells, whereas it did not modify PGI_2_, thus restoring in part, the disequilibrium in the PGI_2_/TXA_2_ ratio observed in cirrhotic HEC. It is plausible to think that NO supplementation only reduced TXA_2_ levels, and not PGI_2,_ due to the increased TXAS expression found in cirrhotic HEC. This effect leads the cell to shift from a vasoconstrictor to a more vasodilatory phenotype. Therefore, NO supplementation in cirrhosis would have a dual beneficial effect, restoring the decreased vasodilator content and attenuating the exaggerated vasoconstrictor production (Fig. 6B). Supporting this concept, it has been demonstrated that simvastatin, a treatment that increases NO availability [26], reduces TXA_2_ release in aged rats [27].

Consistent with our data, it has been observed that other NO-releasing compounds inhibit COX activity in endothelial and smooth muscle cells from other vascular beds [28–31], an effect that has been explained because NO promoted the nitration of tyrosine residues of the COX molecule leading to a loss of enzyme function [13, 32, 33]. By contrast, in different experimental settings a stimulatory effect of NO on prostanoid production through a direct modification of cysteine residues located in the catalytic domain of the COX molecule has been shown [17]. These reported differences have been explained by different cell-specific responses [34] or by variations in the amount of NO [14, 18].

By contrast, NO reduction did not modify COX activity in control or in cirrhotic HEC. This has been previously shown in other endothelial cells, where NOS inhibition did not produce changes in prostanoid production [35] or COX expression [15].

In conclusion, our study demonstrates that NO and COX systems are closely interrelated in HEC. This is particularly relevant in cirrhotic HEC where the release of vasoactive substances is shifted towards vasoconstrictor prostanoids [8]. Our results indicate that in cirrhotic HEC, COX inhibition increases NO bioavailability, and that NO supplementation not only ameliorates the decreased NO availability but also reduces the exaggerated production of TXA_2_, without affecting the release of PGI_2_. This reinforces the rationale to use combined therapies directed to both, increase intrahepatic NO availability and block increased vasoconstrictor prostanoid pathway.
